# Cumulative cancer incidence and mortality after kidney transplantation in Japan: A long‐term multicenter cohort study

**DOI:** 10.1002/cam4.3636

**Published:** 2020-12-13

**Authors:** Ryoichi Imamura, Shigeaki Nakazawa, Kazuaki Yamanaka, Yoichi Kakuta, Koichi Tsutahara, Ayumu Taniguchi, Masataka Kawamura, Taigo Kato, Toyofumi Abe, Motohide Uemura, Tetsuya Takao, Hidefumi Kishikawa, Norio Nonomura

**Affiliations:** ^1^ Department of Urology Osaka University Graduate School of Medicine Suita Osaka Japan; ^2^ Department of Urology Hyogo Prefectural Nishinomiya Hospital Nishinomiya Hyogo Japan; ^3^ Department of Urology Osaka General Medical Center Osaka Japan

**Keywords:** aggressiveness of immunosuppressant regimens, de novo cancer, dialysis duration, kidney transplantation, post‐transplant lymphoproliferative disorders

## Abstract

Kidney transplantation is the most promising treatment to improve mortality and life quality in end‐stage kidney disease; however, cancer remains a leading cause of death. Several factors including immunosuppressants might be associated with a gradual increase in cumulative cancer incidence after kidney transplantation. Risk factors for cancer and overall and cancer‐specific survival were analyzed in 1973 kidney transplant recipients from three study institutions in Japan. The 5‐, 10‐, 20‐, and 30‐year overall and cancer‐specific survival rates were 93.3%, 88.4%, 78.0%, and 63.6% and 99.4%, 98.0%, 95.3%, and 91.7%, respectively. The overall survival rate was significantly higher and the graft survival rate was significantly lower in recipients without cancer than in those with cancer. Older recipient age, longer dialysis duration before kidney transplantation, and history of transfusion were significant predictors of cancer. Dialysis duration before kidney transplantation was a prognostic factor of overall survival rate. Regarding cancer‐specific survival rates, older recipient age and dialysis duration before kidney transplantation were prognostic factors of worse cancer‐specific survival rates. The type of immunosuppressant was not associated with an increased cancer rate. Aggressiveness of immunosuppressant regimens or potent immunosuppressants might improve graft survival rate while inducing de novo cancer after kidney transplantation. Older age and longer dialysis duration before kidney transplantation were risk factors of cancer‐specific survival rate.

## Graphical abstract

Overall patient survival rate was significantly higher in patients without cancer, whereas graft survival rate was significantly higher in those with cancer. The aggressiveness of immunosuppressant regimens or potent immunosuppressant use after kidney transplantation improved graft survival rate, while increasing cancer morbidity. The type of immunosuppressant was not associated with increased cancer morbidity.

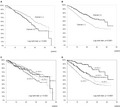

## 1 INTRODUCTION

Kidney transplantation is the most promising treatment that can improve survival and quality of life in patients with end‐stage kidney disease.^1^ While both short‐term patient and graft survival rates after kidney transplants have dramatically improved with the advent of progressive immunosuppressants worldwide,^2^ long‐term survival rate after kidney transplantation has not recovered sufficiently.^3^ Not only immunologic factors such as chronic rejection but also non‐immunologic aspects such as cancer, infection, and cardiovascular disease should be taken into consideration in attempts to improve long‐term survival in these patients.^4^, ^5^ Notably, the incidence and prevalence of de novo cancers are 3–10 folds higher in patients with organ transplants than in the general population.^6^ Even in studies including age‐ and gender‐matched general population cohorts, organ transplant recipients were found to be at increased risk for cancer.^7^, ^8^ Webster et al. have demonstrated that the risk of cancer incidence in a 45‐year‐old transplant patient was equivalent to that of a 70‐year‐old individual in the general population.^8^

Importantly, cancer is a leading cause of death after kidney transplantation.^9^, ^10^ Kidney transplant recipients who develop cancer before graft loss, that is, those with a functioning graft, are at more than 9‐fold risk of death compared to those without cancer, and over 50% of recipients with cancer lose their grafts within 5 years following cancer diagnosis.^10^ Therefore, prevention and early treatment of cancer are essential. In a single‐center study, we previously reported the benefits of cancer screening for early cancer diagnosis.^11^ Specifically, we demonstrated that overall survival rate was significantly lower in kidney transplant recipients without routine cancer screening than in those with cancer screening.

Various factors have been revealed to be associated with cancer after kidney transplantation. The cumulative burden of long‐term immunosuppressive therapy and chronic viral infections is likely to play a crucial role in carcinogenesis after transplantation.^12^ To suppress cancer development, immunosuppressants should be used at lowest doses possible. In fact, several studies demonstrated that lower doses of cyclosporine were associated with reduced cancer incidence compared with the standard dosage.^13^

Additionally, the relationship between kidney transplantation and cancer development should be evaluated specifically for each country or region due to the significant differences in environmental factors such as lifestyle habits and ethnicity. For example, in Western countries, which comprise predominantly Caucasian populations, non‐melanoma skin cancer is the most common post‐transplant cancer in kidney transplant recipients.^12^, ^14^, ^15^ In contrast, urothelial carcinoma is the most common post‐transplant cancer in this patient population in Taiwan, partly because of the use of Chinese herbs.^16^ Conversely, gastric cancer and lymphoma are the most common cancer in Korean patients after kidney transplantation,^17^ whereas non‐Hodgkin lymphoma is the most common cancer reported in patients with kidney transplants in Hong Kong.^18^ These results reflect differences in post‐transplant cancer patterns even among Asian countries.

Moreover, there might be some differences in the influence of oncoviruses on cancer development in patients among different countries. Kaposi sarcoma, one of the high standardized incidence ratio for cancers in kidney transplant recipients, is caused by human herpes virus 8, but the sarcoma is extremely rare in Japan. Therefore, examining the prevalence of de novo cancers after kidney transplantation in specific countries and regions is vital. The present study aimed to investigate cancer trends and cancer‐specific and all‐cause mortality in kidney transplant recipients with comparison to those without cancer incidence in Japan and discuss future measures to prevent cancer development in this vulnerable patient population.

## 2 MATERIALS AND METHODS

### 2.1 Recipient characteristics

We retrospectively reviewed the medical records of 1973 kidney transplant recipients who underwent kidney transplantation in Osaka University Hospital (*n* = 910), Hyogo Prefectural Nishinomiya Hospital (*n* = 648), and Osaka General Medical Center (*n* = 415) between June 1965 and September 2019. Data of all recipients were extracted using the REDCap^®^ software (Vanderbilt University), an electronic registration system. Demographic characteristics and relevant information such as transplant and cancer history, immunosuppressant regimens, dialysis duration before kidney transplantation, renal allograft conditions including rejection, viral infections, lifestyle habits, and history of transfusion, pregnancy, and comorbidities were collected. The collected data were analyzed to investigate patient characteristics, patient survival rate, graft survival rate, cumulative cancer incidence rate, and cancer types.

Induction immunosuppression therapies included calcineurin inhibitors (cyclosporine and tacrolimus), metabolic nucleic acid inhibitors (azathioprine, mizoribine, and mycophenolate mofetil [MMF]), and steroids. Additionally, antilymphocyte globulin (used from 1993 to 2003) or anti‐CD25 antibody (basiliximab, used from 2004 to 2019) was added for induction therapy. The target trough levels of cyclosporine were 250–300 ng/ml up to 30 days after kidney transplantation, 150–200 ng/ml (postoperative day [POD] 31–60), and 50–120 ng/ml (after POD 61). The target trough levels of tacrolimus (‐2000) were 15–20 ng/ml for 14 days after transplantation, and then gradually decreased to 5–10 ng/ml. After the combined use of MMF (2001‐), the target value was 5–10 ng/ml for 1 year after transplantation, and gradually decreased to 4–5 ng/ml after the 1st year. Azathioprine was administered at 50 or 100 mg/day. MMF was 2000 mg/day for 2 weeks after kidney transplantation, gradually decreased, and 1000 mg/day was administered from the 3rd week. In 2012, a mammalian target of rapamycin inhibitor (mTORi, target trough; 3.0–8.0 ng/ml) was initiated as additional induction therapy in some patients. For ABO blood‐type incompatible kidney transplantation, splenectomy or rituximab infusion was performed. A kidney biopsy was performed in patients with elevated serum creatinine levels. For biopsy‐proven rejection, methylprednisolone was administered for 3 days. The same approach was used in patients who could not be evaluated by kidney biopsy but were clinically diagnosed with rejection (e.g., over 20% elevation in serum creatinine level). In patients with steroid‐resistant rejection, gusperimus hydrochloride or an anti‐CD3 monoclonal antibody was used after methylprednisolone treatment. Starting in 2011, thymoglobulin was used as an alternative therapy for T‐cell–mediated rejection. For antibody‐mediated rejection, plasma exchange, rituximab infusion, and intravenous immunoglobulin therapy were used. All procedures were performed in accordance with the 1975 Helsinki Declaration. This study protocol was approved by the ethics committees of Osaka University Hospital (approval number: 19475), Hyogo Prefectural Nishinomiya Hospital (approval number: H28‐19), and Osaka General Medical Center (approval number: 28‐2034).

### 2.2 Statistical analysis

Categorical variables were presented as percentages or frequencies, whereas continuous variables were presented as means with standard deviation or medians with interquartile range. In the present study, the transplant recipients were categorized into cancer‐positive and cancer‐negative groups and to investigate risk factors of de novo cancer development after kidney transplantation. Differences in characteristics between the two groups were compared. In addition, graft survival and overall survival rates were compared between not only these two groups but also four groups, cancer‐positive or ‐negative/rejection‐positive or ‐negative. Univariate analyses were performed using the Mann–Whitney, Kruskal–Wallis, chi‐square, and Fisher's exact tests to compare continuous and categorical variables, as appropriate. The Kaplan–Meier method with the log‐rank test and multiple comparison tests was used to compare patient and graft survival rates. The Gray's test was used to compare the cumulative cancer incidence rate. Cox proportional hazard regression analysis was used to determine hazard ratios (HRs) for cancer development, overall survival, and cancer‐specific survival. Cox regression analysis was also used to assess prognostic factors preventing post‐transplant cancer. The statistical significance level was defined as two‐tailed *p* < 0.05.

## 3 RESULTS

### 3.1 Differences in overall patient and graft survival rates between cancer‐positive and cancer‐negative groups

All patient characteristics are summarized in Table 1. Among a total of 1973 recipients, 241 developed cancer, including 31 recipients who developed two primary cancers and 3 recipients who developed three primary cancers after transplantation, whereas the remaining 1732 recipients did not develop cancer during the study period. The mean interval between kidney transplantation and the time of cancer diagnosis was 12.8 ± 8.5 years, and the mean age of diagnosis was 52.2 ± 11.8 years. The mean dialysis duration before kidney transplantation was significantly longer in the cancer‐positive group than in the cancer‐negative group (4.96 ± 5.88 years vs. 4.03 ± 5.62 years, *p* < 0.001). The rates of transplantation from a deceased donor and ABO blood‐type compatible kidney transplantation were significantly higher in the cancer‐positive group than in the cancer‐negative group (22.8% vs. 17.0%, *p* = 0.030, and 90.9% vs. 86.1%, *p* = 0.032, respectively). Moreover, the rates of patients with a history of rejection and those with a history of transfusion were significantly higher in the cancer‐positive group than in the cancer‐negative group (54.4% vs. 42.1%, *p* < 0.001 and 27.4% vs. 20.3%, *p* = 0.014, respectively). The rate of rituximab use was higher in the cancer‐negative group than in the cancer‐positive group (11.5% vs. 4.56%, *p* < 0.001).

**TABLE 1:** Demographic characteristics of transplantation recipients included in the study

Factors	Cancer (+) (*n* = 241)	Cancer (−) (*n* = 1732)	*p*
Recipient gender (female/male)	104/137 (43.2/56.8)	652/1080 (37.6/62.4)	0.101
Recipient age at transplantation (years)	39.4 (12.5)	38.9 (14.0)	0.82
Donor gender (female/male)	132/109 (45.2/54.8)	1062/670 (61.3/38.7)	0.053
donor age at transplantation (years)	51.5 (13.5)	52.5 (12.7)	0.472
History of prior transplant (yes/no)	13/228 (5.4/94.6)	72/1660 (4.2/95.8)	0.374
Recipient BMI (kg/m^2^)	21.4 (1.86)	21.3 (2.21)	0.3
Dialysis duration before kidney transplantation (years)	2.38 [1.00, 6.90]	1.75 [0.58, 5.00]	<0.001
Donor type (living/deceased)	186/55 (77.2/22.8)	1438/294 (83.0/17.0)	0.030
ABO blood type (compatible/incompatible)	219/22 (90.9/9.1)	1491/241 (86.1/13.9)	0.032
Blood relative (yes/no)	163/78 (67.6/32.4)	1157/575 (66.8/33.2)	0.825
HLA mismatches (*n*)	2.20 (1.40)	2.19 (1.52)	0.952
History of rejection (%)	54.4	42.1	<0.001
Smoking habit (%)	4.56	8.40	0.028
HCV antibody positive (%)	3.73	1.84	0.078
Pre‐transplant diabetes (%)	8.71	12.6	0.073
History of transfusion (%)	27.4	20.3	0.014
History of pregnancy (%)	2.90	2.54	0.743
Rituximab for induction treatment (%)	4.56	11.5	<0.001
Splenectomy prior to transplant (%)	6.22	5.14	0.490
Utilization of CNI (none/CyA/TAC, %)	17.0/49.4/33.6	12.8/38.2/49.0	<0.001
Utilization of MI (none/AZA/MZ/MMF, %)	2.50/45.6/17.8/34.0	3.7/28.9/16.4/51.0	<0.001
Observation period (years)	19.1 (10.3)	12.6 (10.3)	<0.001

Note

Categorical variables are presented as frequencies and/or percentages, and continuous variables are presented as means with standard deviation. Dialysis duration before kidney transplantation is presented as median with interquartile because of non‐normal distribution.

Abbreviations: AZA, azathioprine; BMI, body mass index; CNI, calcineurin inhibitor; CyA, cyclosporine A; HCV, hepatitis C virus; MI, metabolic inhibitor; MMF, mycophenolate mofetil; MZ, mizoribine; TAC, tacrolimus.

**FIGURE 1:**
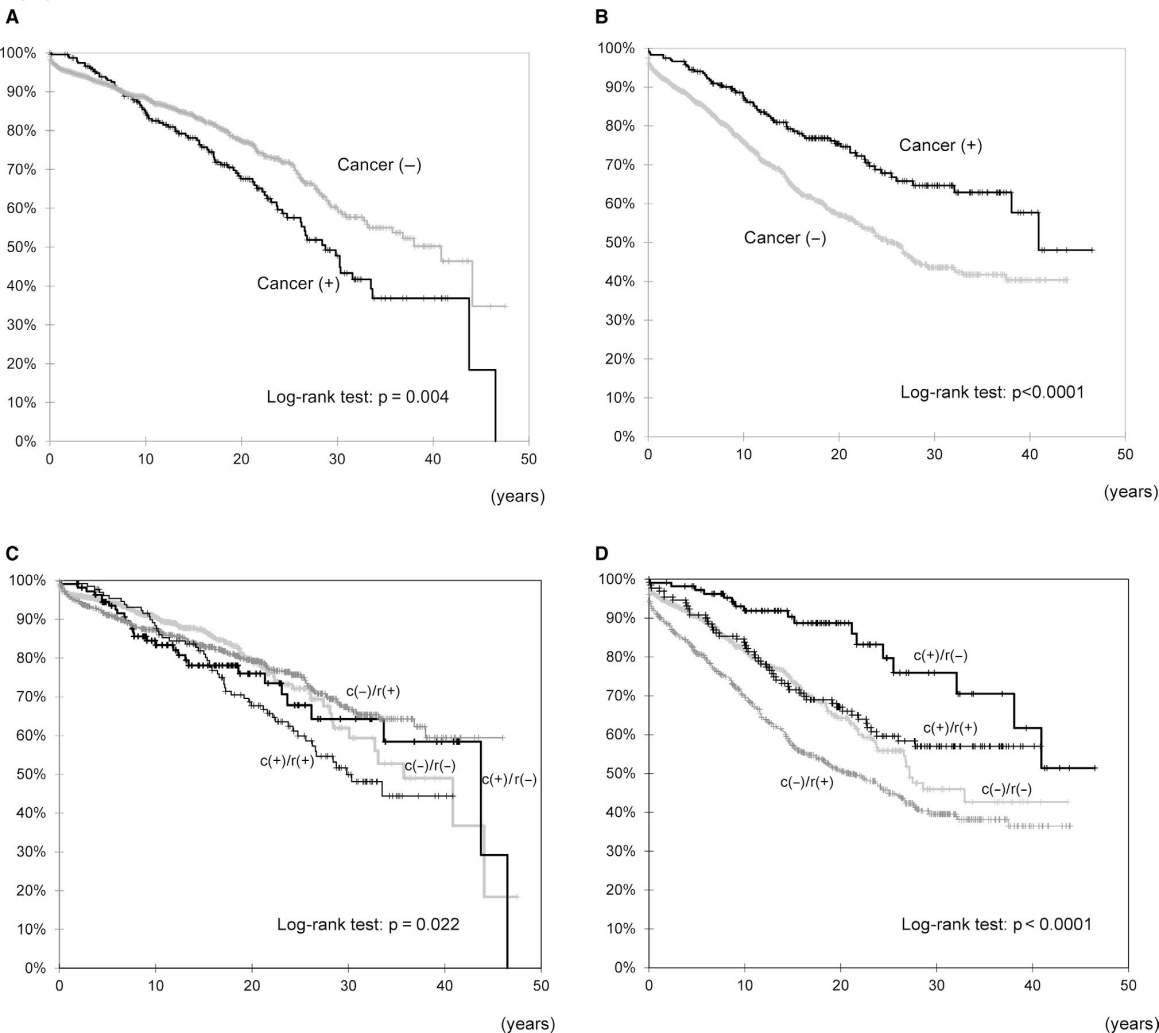
Comparison of survival between patients with and without cancer. (A) Patient survival rate and (B) graft survival rate. Black line: with cancer, gray line: without cancer. Patient survival rate (C) and graft survival rate (D) classified by the presence or absence of cancer and rejection. Black thick line: cancer‐positive/rejection‐negative, Black thin line: cancer‐positive/rejection‐positive, dark gray line: cancer‐negative/rejection‐positive, light gray line: cancer‐negative/rejection‐negative

Comparison of the overall patient and graft survival rates between the cancer‐positive and cancer‐negative groups revealed that the overall patient survival rate in the cancer‐positive group was significantly lower than that in the cancer‐negative group (*p* = 0.004, log‐rank test, Figure 1A). Specifically, the 5‐, 10‐, and 20‐year patient survival rates were 95.2%, 85.7%, and 70.4%, respectively, in the cancer‐positive group and 93.0%, 88.9%, and 79.9%, respectively, in the cancer‐negative group. In contrast, the graft survival rate was significantly higher in the cancer‐positive group than in the cancer‐negative group (*p* < 0.001, log‐rank test, Figure 1B). The 5‐, 10‐, and 20‐year graft survival rates were 94.0%, 87.5%, and 76.3%, respectively, in the cancer‐positive group and 86.2%, 75.8%, and 56.9%, respectively, in the cancer‐negative group. Therefore, the overall patient survival rate was significantly higher in the cancer‐negative group, whereas the graft survival rate was significantly higher in the cancer‐positive group. To examine the results, we further classified these two groups into additional two groups, one with rejection (rejection‐positive) and one without rejection (rejection‐negative), and compared the patient (Figure 1C) and graft (Figure 1D) survival rates in the four groups (Log‐rank test, *p* = 0.022). The patient survival rate was significantly lower in the cancer‐positive/rejection‐positive group than in the other groups. On the other hand, the graft survival rate was significantly higher in the cancer‐positive/rejection‐negative group, equivalent in the cancer‐positive/rejection‐positive group and cancer‐negative/rejection‐negative group, and significantly lower in the cancer‐negative/rejection‐positive group (Log‐rank test, *p* < 0.0001).

### 3.2 Overall and cancer‐specific survival rates of the entire study cohort

**FIGURE 2:**
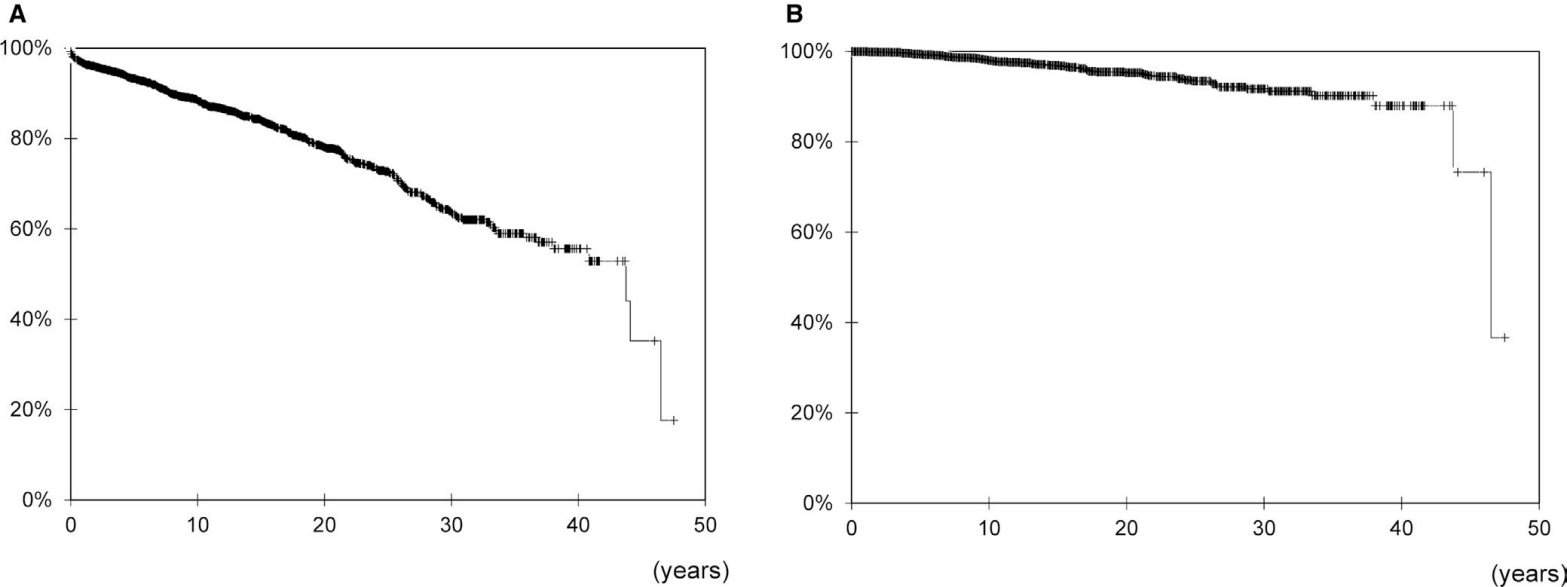
Patient survival rates after kidney transplantation. (A) Overall survival and (B) cancer‐specific survival

In the study cohort of 1973 recipients, 5‐, 10‐, 20‐, and 30‐year overall survival rates were 93.3%, 88.4%, 78.0%, and 63.6%, respectively (Figure 2A), and 5‐, 10‐, 20‐, and 30‐year cancer‐specific survival rates were 99.4%, 98.0%, 95.3%, and 91.7%, respectively (Figure 2B).

### 3.3 Cancer types and cumulative de novo cancer incidence rate after kidney transplantation

**FIGURE 3:**
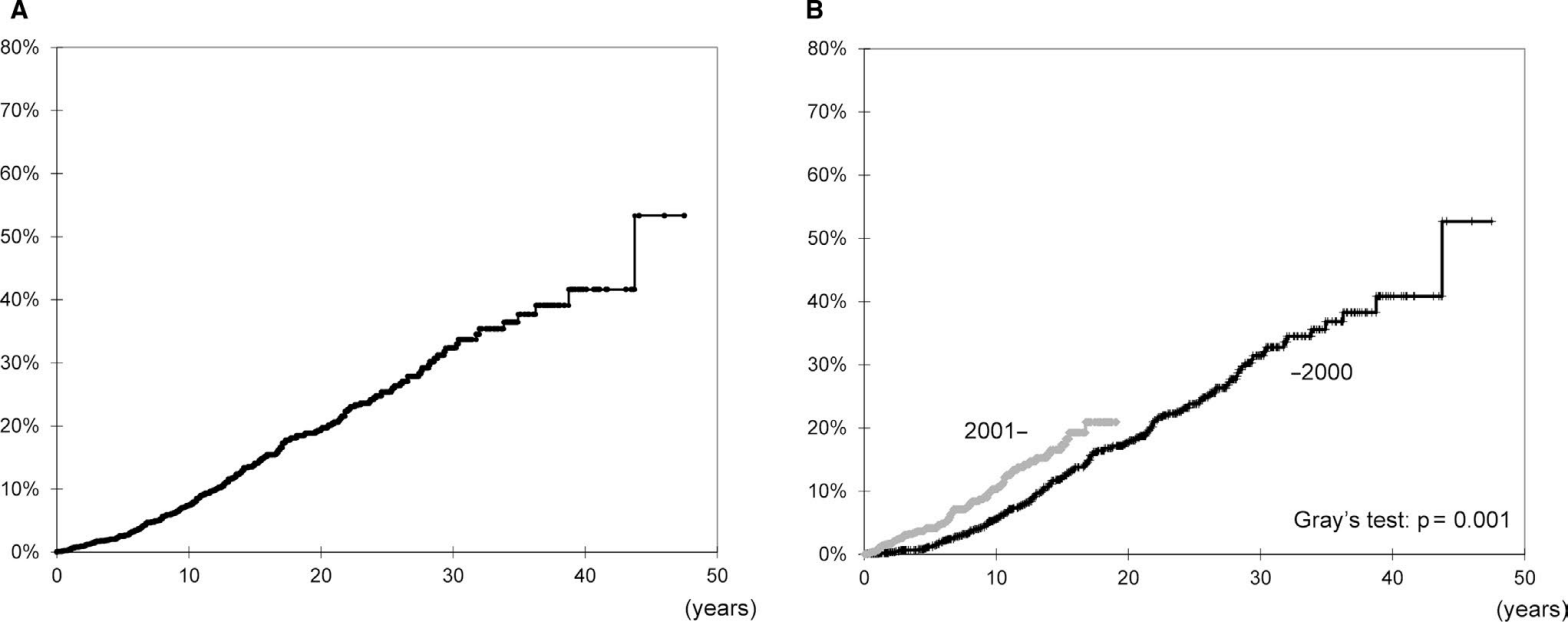
Total cumulative cancer incidence after kidney transplantation (A) and the cumulative morbidity of the two groups until 2000 (black line) and after 2001 (gray line) (B)

The types of 278 cancers diagnosed in the study cohort are summarized in Table 2. The most frequently detected cancer types were post‐transplant lymphoproliferative disorders (PTLD, *n* = 37), skin cancer (*n* = 32), breast cancer (*n* = 28), and renal cell carcinoma of original kidney (*n* = 28). From 2001, we have changed the immunosuppressive regimen dramatically (decreasing target trough level of CNI and metabolic nucleic acid inhibitors to MMF). Therefore, we have listed the number of cancers detected in each period (‐2000 and 2001‐) individually. The most frequently detected cancer types in both groups were PTLD (*n* = 24 and 13, respectively). As shown in Figure 3A, the cumulative de novo cancer incidence rates at 5, 10, and 20 years were 2.5%, 7.5%, and 19.4%, respectively. Moreover, we compared the cumulative morbidity of the two groups, until 2000 and after 2001 (Figure 3B). The cumulative morbidity rate was significantly higher in the group after 2001 (Gray's test, *p* = 0.001).

**TABLE 2:** Cancer types diagnosed in 241 recipients

Type of cancer	*n*		
Implementation period of transplantation	Total	‐2000	2001‐
PTLD	37	24	13
Skin cancer	32	23	9
Breast cancer	28	18	10
Renal cell carcinoma	28	17	11
Colorectal cancer	18	13	5
Gastric cancer	18	13	5
Uterus cancer	15	10	5
Prostate cancer	13	5	8
Hepatocellular carcinoma	12	12	0
Tongue cancer	12	10	2
Urothelial cancer	12	8	4
Thyroid cancer	10	6	4
Pancreas cancer	4	2	2
Lung cancer	2	2	0
Ovarian cancer	2	2	0
Anal cancer	1	1	0
Vaginal cancer	1	1	0
Others	33	25	8
Total	278	192	86

Abbreviation: PTLD, post‐transplant lymphoproliferative disorders.

### 3.4 Cox proportional hazard regression analysis for cancer development and survival

Cox proportional hazard regression analyses were performed to clarify risk factors of the cancer development and prognostic factors of recipients who developed cancers after kidney transplantation. By multivariate analysis, older recipient age (HR 1.033, 95% confidence interval [CI] [1.019, 1.046], *p* < 0.001), longer dialysis duration before kidney transplantation (HR 1.024, CI [1.001, 1.049], *p* = 0.043), and history of transfusion (HR 1.427, CI [1.053, 1.934], *p* = 0.022) were significant predictors of cancer development (Table 3). Additionally, by multivariate analysis only longer dialysis duration before kidney transplantation (HR 1.025, CI [1.004, 1.046], *p* = 0.019) was a prognostic factor for overall survival rate (Table 4), whereas older recipient age (HR 1.032, CI [1.009, 1.055], *p* = 0.007) and longer dialysis duration before kidney transplantation (HR 1.048, CI [1.007, 1.091], *p* = 0.023) were prognostic factors for cancer‐specific survival rate (Table 5). The type of immunosuppressant was not associated with increased cancer morbidity.

**TABLE 3:** Cox proportional hazard regression analysis of risk factors for cancer development

Clinical factors	Univariate analysis				Multivariate analysis			
	Hazard ratio	95% confidence interval		*p* value	Hazard ratio	95% confidence interval		*p* value
Recipient age	1.033	1.019	1.046	<0.001	1.033	1.019	1.046	<0.001
Recipient gender	0.982	0.760	1.269	0.892				
Dialysis duration (years)	1.052	1.030	1.074	<0.001	1.024	1.001	1.051	0.043
History of rejection	0.861	0.666	1.115	0.257				
History of transplantation	2.136	1.217	3.746	0.008	1.478	0.826	2.644	0.188
Donor type (living/deceased)	0.760	0.563	1.026	0.073				
History of transfusion	1.523	1.147	2.022	0.004	1.427	1.053	1.934	0.022
History of pregnancy	2.100	1.107	3.983	0.023	1.770	0.817	3.833	0.148
History of smoking habit	2.189	1.173	4.085	0.014	1.658	0.864	3.185	0.129
Blood relative	0.667	0.000	0.875	0.003	1.203	0.710	2.038	0.492
Cyclosporine	1.252	0.863	1.816	0.237				
Tacrolimus	1.579	1.049	2.376	0.028	1.240	0.756	2.035	0.394
Azathioprine	1.416	0.622	3.224	0.407				
Mycophenolate mofetil	2.207	0.955	5.100	0.064				
Rituximab	1.321	0.714	2.443	0.376				

**TABLE 4:** Cox proportional hazard regression analysis of prognostic factors for overall survival in patients with post‐transplant cancer

Clinical factors	Univariate analysis				Multivariate analysis			
	Hazard ratio	95% confidence interval		*p* value	Hazard ratio	95% confidence interval		*p* value
Recipient age	1.038	1.018	1.058	<0.001	1.009	0.999	1.019	0.065
Recipient gender	1.302	0.849	1.997	0.227				
Dialysis duration (years)	1.062	1.026	1.099	0.001	1.025	1.004	1.046	0.019
Blood relative	0.611	0.000	0.942	0.026	0.942	0.724	1.227	0.659
History of transfusion	1.150	0.901	1.468	0.263				
History of smoking habit	1.408	0.342	5.803	0.636				
Cyclosporine	0.856	0.520	1.409	0.540				
Tacrolimus	0.891	0.470	1.690	0.724				
Azathioprine	3.755	0.520	27.122	0.190				
Mycophenolate mofetil	3.863	0.509	29.323	0.191				
Rituximab	3.638	1.297	10.21	0.014	0.582	0.316	1.071	0.082

**TABLE 5:** Cox proportional hazard regression analysis of prognostic factors for cancer‐specific survival in patients with post‐transplant cancer

Clinical factors	Univariate analysis				Multivariate analysis			
	Hazard ratio	95% confidence interval		*p* value	Hazard ratio	95% confidence interval		*p* value
Recipient age	1.025	1.002	1.049	0.035	1.032	1.009	1.055	0.007
Recipient gender	0.941	0.569	1.558	0.814				
Dialysis duration (years)	1.060	1.017	1.104	0.005	1.048	1.007	1.091	0.023
Blood relative	0.884	0.508	1.539	0.663				
History of smoking habit	0.957	0.131	6.999	0.965				
Cyclosporine	0.797	0.445	1.427	0.445				
Tacrolimus	0.519	0.227	1.184	0.119				
Azathioprine	2.622	0.360	19.081	0.341				
Mycophenolate mofetil	2.258	0.288	17.691	0.438				
Rituximab	1.121	0.153	8.226	0.911				

## 4 DISCUSSION

In the present study, examining risk factors associated with de novo cancer development as well as the rates of graft survival, overall survival, and cancer‐specific survival after kidney transplantation, we surprisingly found that the graft survival rate of the cancer‐positive group was better than that of the cancer‐negative group. Multivariate analysis by Cox proportional hazards model revealed that none of the immunosuppressants was associated with increased cancer morbidity. Therefore, we speculated that the aggressiveness of the immunosuppressive regimen after kidney transplantation improved graft survival rate while increasing cancer morbidity, as Opelz et al.^19^ described previously. The graft survival rate of the cancer‐positive/rejection‐negative group was significantly higher than other groups, and the rate of the cancer‐positive/rejection‐positive group was similar to that of the cancer‐negative/rejection‐negative group. We regularly perform cancer screening for all recipients once a year.^11^ Most of the patients classified as cancer‐positive/rejection‐negative could be detected cancers in the early stage by the screening and could be treated without reducing the dose of immunosuppressant even if they received aggressive immunosuppressive therapy (data not shown). On the other hand, most of the patients in the cancer‐positive/rejection‐positive group consisted of the recipients (1) who had not undergone cancer screening, (2) who could not be detected by the cancer screening, and (3) whose immunosuppressive therapy was enhanced by additional treatment due to acute rejection. Whichever comes first, aggressive immunosuppression and rejection, the balance between them was predicted to significantly impact cancer morbidity and graft survival rate. In these points, further studies will be required in the future.

In the early period after kidney transplantation, the patient survival rate declined due to diseases other than malignant neoplasms, such as infectious and cardiovascular diseases (data not shown). On the other hand, approximately 10 years after kidney transplantation, the survival rate of cancer‐positive decreased. We have considered devising countermeasures and found that the survival rate was the worst in the cancer‐positive/rejection‐positive group in Figure 1C. These results indicated that it was important to detect and treat cancer in an early stage, as mentioned above.

We treated with certain regimens in each era. But due to the range of target troughs (cyclosporine, tacrolimus, mTORi) and the fixed amount of administration that did not consider individual differences (MMF, rituximab), it could not be denied that the regimen might have become aggressive for some recipients. In the previous report, for example, Dantal et al. compared two groups of recipients receiving cyclosporine for maintenance immunosuppression, target trough levels of <125 ng/ml vs. >150 ng/ml.^13^ They found that the rate of cancer development rate was lower in the low‐dose group than in the high‐dose group. Additionally, long‐term and strong immunosuppression is proposed to induce several alterations in immunity and immune phenotype in kidney transplant recipients, which can subsequently lead to cancer development through weakened tumor surveillance.^20^, ^21^, ^22^ Based on the accumulating evidence, it was suggested that the dose rather than the type of immunosuppressants used for induction therapy might be associated with cancer development in kidney transplant recipients. In our results (Table 1), rituximab usage rate was significantly higher in cancer‐negative group. Currently, only preoperative administration for ABO blood type incompatible transplantation is permitted in Japan. Therefore, the number of cases and the observation period are not sufficient. It will be necessary to accumulate data and perform further analysis.

Previously, we also reported that age at the time of transplantation and treatment with tacrolimus were significant risk factors for cancer development by multivariate analysis and concluded that the incidence of cancer would further increase if the patients administered tacrolimus were followed for longer periods, which would reveal the substantial effect of tacrolimus on outcomes of renal transplant recipients.^23^ However, tacrolimus was not a risk factor of cancer development in the current study. The average tacrolimus induction dose used at kidney transplantation was 15–20 ng/mL in patients included in our previous study. Currently, tacrolimus is routinely used for kidney transplantation; however, the average induction dose is less than 7 ng/ml and less than 5 ng/ml tacrolimus is prescribed at 1 year after transplantation with the introduction of new generation of immunosuppressants such as MMF, basiliximab, and mammalian target of rapamycin inhibitor. We speculate that the low tacrolimus dose for induction and maintenance immunosuppression might have contributed to the reduced risk of cancer development. The cumulative cancer incidence of our group was lower than that reported from other countries.^24^, ^25^, ^26^ For example, in Europe, a tacrolimus dose above 6.3 ng/ml is recommended for maintenance immunosuppression to prevent the generation of donor‐specific antibodies that can induce antibody‐mediated rejection.^27^ Additionally, thymoglobulin, a T‐cell–depleting agent, has been increasingly used for induction therapy immediately after kidney transplantation in other countries but not in Japan.^28^ Induction treatment with T‐cell–depleting agents has been reported to increase the risk of lymphoma by 20‐fold, compared with the age‐ and gender‐matched general population.^29^ These factors might have contributed to the observed differences in cumulative cancer incidence reported from other countries and our group. Regarding cancer development, it may be necessary to fully consider induction therapy for all patients undergoing kidney transplantation except for some immunological high‐risk patients in the future.

In Japan, for the general population, colorectal cancer, gastric cancer, and lung cancer are the most prevalent cancers. Also, prostate cancer and breast cancer have the highest prevalence in men and women, respectively.^30^ The general population and kidney transplant recipients had different tendencies for the type of cancer they had. For PTLD and skin cancer, oncoviruses (Epstein‐Barr virus and human papillomavirus, respectively) infections, duration and level of immunosuppressants are known to be important.^31^, ^32^ Therefore, it was considered that we should perform cancer screening before kidney transplantation and once a year (at least once every few years) after the transplantation for early diagnosis.

With multivariate analysis by Cox proportional hazard regression model, we found that a history of transfusion increased the risk of cancer development. It is known to induce the production of antibodies, including donor‐specific antibodies, through sensitization in the recipient. With the advent of modern potent immunosuppressants, kidney transplantation can be performed even in recipients with risk factors of acute rejection, such as those with preformed donor‐specific antibodies. The immunosuppressant dose might be purposefully increased to prevent rejection due to these antibodies. Therefore, it remains possible that these factors might eventually lead to the development of cancer.

We investigated risk factors of overall survival and cancer‐specific survival using the Cox proportional hazard regression analysis. Dialysis duration before kidney transplantation was a risk factor not only for overall survival but also for cancer‐specific survival. The correlation of time on dialysis with the risk of cancer development, which has been already reported,^33^ might have contributed to the observed role of dialysis duration as a risk factor for cancer‐specific survival. Additionally, infectious diseases, cardiovascular diseases, and cancer are three known major causes of death after kidney transplantation regardless of patient age in Japan^34^ as well as in other countries.^35^ One study reported that the prevalence rates of infectious disease and cardiovascular disease increased with increasing dialysis vintage.^36^ Therefore, we speculate that these comorbidities might have contributed as prognostic factors of overall survival after transplantation as well.

The present study has several notable strengths. The study was based on the largest population of kidney transplant recipients with the longest study duration and with very little missing baseline data. The completeness of the dataset suggests that selection and ascertainment biases in exposure and study factors are minimal. However, the present study has several limitations that should also be acknowledged. First, this was a retrospective multicenter study including three institutions. Second, despite adjustment for all confounding factors, there may be unmeasured residual effects such as dose and duration of immunosuppressants used for the treatment of primary disease and incomplete details on smoking habits and alcohol consumption, which may have altered the strength and magnitude of association of cancer risk after transplantation. Finally, cancer treatment approaches and reduction in immunosuppressant doses in patients who developed cancer were determined by the attending physician in each patient. However, despite these limitations, the current study findings are important as they are based on the largest Japanese cancer registry data of patients with kidney transplants.

In conclusion, the aggressiveness of immunosuppressant regimens or potent immunosuppressant use might improve graft survival rate, while increasing the risk of de novo cancer after kidney transplantation. Older recipient age, dialysis duration before kidney transplantation, and risk factors of rejection (e.g., history of transfusion) were significant factors associated with cancer development. To improve overall and cancer‐specific survival rates, it is essential to reduce immunosuppression to the greatest extent possible in older recipients. Additionally, patients with end‐stage kidney disease should undergo kidney transplantation before the induction of dialysis or, at least, as soon as possible if they consider undergoing kidney transplantation. It was suggested that cancer screening, including computed tomography scan, gastrointestinal examination, and dermatological consultation, should be performed regularly, at least 10 years after kidney transplantation.

## CONFLICT OF INTEREST

All authors declare that there are no conflicts of interest.

## Data Availability

The data that support the findings of this study are available on request from the corresponding author. The data are not publicly available due to privacy or ethical restrictions.
